# Clinicopathological Comparison of Adenocarcinoma of Cervix and Endometrium Using Cell Cycle Markers: P16ink4a, P21waf1, and p27Kip1 on 132 Cancers

**DOI:** 10.1155/2011/857851

**Published:** 2011-10-24

**Authors:** Farveen Marican Abu Backer, Nik Raihan Nik Mustapha, Nor Hayati Othman

**Affiliations:** ^1^Pathology Department, Hospital Universiti Sains Malaysia (HUSM), Kelantan, Malaysia; ^2^Pathology Department, Hospital Sultanah Bahiyah (HSB), Alor Star, Malaysia

## Abstract

*Objective*. We studied the clinicopathological parameters of adenocarcinoma arising from endocervix (ECA) and from endometrium (EMA) based on the expression of P16ink4a, P21waf1, and p27Kip1 proteins. *Study Design*. Immunohistochemistry was done on sections of confirmed ECA and EMA from hysterectomy specimens which have had no prior chemotherapy/radiotherapy. *Results*. There were 40 ECAs and 92 EMAs. The mean age of ECA was 49.82 (SD 10.29); the youngest was 30 years old and the oldest 75 years old. The mean age of EMA was 54.45 (SD 10.92); the youngest was 30 years old and the oldest was 82 years old. For ECA, the size of the tumour is significantly associated with age and with depth of infiltration. FIGO stage is associated with histological grade. p21WAF1 expression is significantly associated with infiltration of the corpus and lymph node metastasis. p27Kip1 expression is significantly associated with lymph node invasion. The presence of lymph node metastasis is strongly associated when p16INK4a and p27Kip1 expressions are analyzed in combination. For EMA, p16INK4a expression is associated with histologic grade. *Conclusion*. Our study shows that we could use these cell cycle markers as predictors for more aggressive subsets of adenocarcinoma of the cervix and endometrium.

## 1. Introduction

The trend of adenocarcinoma of the cervix (ECA) is increasing [1–3] particularly among younger women [4]. Adenocarcinoma of the endometrium on the other hand affect older women, in the fifth to six decade [5]. In routine clinical practice, the presentation of these two cancers often overlap [6] and each requires different management [7]. ECA has more risk of recurrence [8] than endometrial adenocarcinoma. In Malaysia, adenocarcinoma of the cervix accounts for 5–15% of the total cervical cancers and the trend is also increasing [9]. The histological appearance of these two cancers under routine H/E staining is almost similar in many instances. In a study on fractional curettage specimens from fifteen women who had tumours in the endocervical and endometrial specimens, only 34.1% were diagnosed as endometrial carcinomas [10]. Carcinoembryonic antigen (CEA), estrogen receptor (ER), vimentin, and a panel of histochemical stains [11] are routinely used to differentiate these two cancers [12, 13]. Routine IHC studies may not be helpful for synchronous endometrial and endocervical tumors [14]. In such, clonality studies using robust molecular techniques may help diagnose cases in which conventional imunohistochemical studies are not helpful [14]. 

p16INK4A is a molecular biomarker that consistently discriminates uterine cervix adenocarcinoma from endometrial adenocarcinoma [15]. It is a cell cycle regulatory tumour suppressor gene that has intimate interplay with the retinoblastoma gene [16]. p16INK4A could differentiate similar skin conditions: actinic keratosis from Bowens disease of which differentiating them before definitive treatment is important as each has different management [16]. p21WAF1 is a cyclin-dependent kinase inhibitor which acts as both a sensor and an effector of multiple antiproliferative signals [17]. p27Kip1 is a negative regulator of the G1 phase of the cell cycle [18]. Attempts at differentiating the clinicopathological parameters of adenocarcinoma of the cervix from endometrium have been done by a number of researchers using immunohistochemical stains [13, 14] and molecular techniques such as Western blotting [19] and PCR [20, 21]. Among the parameters studied was presence of estrogen receptor status [19, 22].

We studied the clinical parameters of adenocarcinomas arising from endocervix based on the expression of P16ink4a, P21waf1, and p27Kip1 proteins and whether the presence/absence of such protein expression correlate with clinical presentations/behaviour of these cancers. 

## 2. Methodology

This is a retrospective study on archived tissue blocks of histologically confirmed adenocarcinoma of the cervix and endometrium diagnosed from 2005 to 2008. The samples were hysterectomy specimens with or without removal of ovaries and fallopian tubes. Biopsies (either colposcopic or by curettage) were excluded. Specimens from patients who have had chemotherapy or radiotherapy before hysterectomy were excluded. These samples were taken from two public hospitals located in two different localities: Hospital Universiti Sains Malaysia (HUSM) (north-eastern region) and Hospital Sultanah Bahiyah (HSB) (north-western region of Malaysia). 

The demography and the clinical details were obtained from the case folders. The histological diagnoses of ECA and EMA were categorized according to the main histological feature as endocervical NOS, adenosquamous, endometrioid, serous, and clear cell. The cancers are called “mixed” when the histological findings show more than one histological patterns. Immunohistochemistry staining was done on 4 micron thickness sections from paraffin blocks using the standard immunohistochemical staining technique. p16INK4a, P21WAF1, and P27Kip1 primary antibodies were at 1 : 100 dilutions. Positive controls were squamous cell carcinoma for p16INK4a, colonic adenocarcinoma for p21KIP1, and normal prostatic tissue for p27Kip1 tissue as per suggestion by manufacturers of these antibodies. The association was determined using multiple logistic regression tests. The results were analysed using SPSS version 16, and the level of confidence (*P* value) was set at 0.05.

The study was approved by the Research Ethics Committee of Universiti Sains Malaysia.

## 3. Results

There were 40 adenocarcinomas (ECAs) of the endocervix and 92 adenocarcinomas of the endometrium (EMAs). The median age for adenocarcinoma of the cervix was 48.5 years, and the mean age was 49.82 (SD 10.3). The youngest patient was 30 years old and the oldest 75 year old. The median age for adenocarcinoma of the endometrium was 55.0 years and the mean age was 54.45 (SD 10.9). The youngest patient was 30 years old and the oldest 82 years old. For adenocarcinoma of the cervix, there were 74% Malays, 18% Chinese, 8% Siamese, and no Indian patients while for endometrial carcinoma, there were 79% Malays, 11% Chinese, 9% Indian, and 1% Siamese ethnic group. 

The most common histological type of adenocarcinoma of the cervix was mucinous type: 62.5% (25/40), and the commonest histological subtype of endometrial adenocarcinoma was endometrioid type (75/92). Majority of the patients were in FIGO stage I (ECA 57.5%, EMA 54.3%). The summary of the clinicopathological findings of the 132 cancers is in Table 1. 

The interrelationship between the various clinicopathological correlations is as shown in Table 2 for ECA and Table 3 for EMA. For adenocarcinoma of the cervix, the grade was significantly associated with histologic type and with lymph node. The age of the patients was significantly associated with the size of the tumor. The tumor size is also associated with the depth of infiltration. FIGO stage is strongly associated with vascular and lymph node invasion (Table 2). For adenocarcinoma of the endometrium, FIGO stage is associated with histological grade and expectedly with myometrial invasion (Table 3). 

For adenocarcinoma of the cervix, none of the clinicopathological parameters is significantly associated with p16INK4a expression (Table 4) including the histologic subtypes. p21WAF1 expression (Table 5) is significantly associated with infiltration of the corpus (*P* = 0.043) and lymph node metastasis (*P* = 0.071), and p27Kip1 expression (Table 6) is significantly associated with lymph node invasion (*P* = 0.030). The presence of lymph node metastasis is strongly associated (*P* = 0.013) when p16INK4a and p27Kip1 expressions are analyzed in combination. 

For adenocarcinoma of the endometrium, p16INK4a expression (Table 7) is associated with histologic grade (*P* = 0.014) but not the histologic type (*P* = 0.888). p21WAF1 and p27Kip1 did not show significant associations with clinicopathologic parameters (*P* > 0.05). 

## 4. Discussion

Adenocarcinoma arising from the cervix and the endometrium in our series shows overlapping clinicopathological presentations. The youngest patients for both cancers were 30 years of age though the mean age of endocervical adenocarcinoma is about five years younger. There was a number of adenocarcinomas of the cervix which was in the mean age of adenocarcinoma of endometrium. These two cancers also have similar ethnic distribution. The ethnic distribution of our cases is comparable with the background population implying fair representation of the sampling population. They are more seen among the ethnic Malay than other ethnic groups in this series depicting the ethnic distributions of the community served by the two hospitals. 

We noted that certain clinicopathological parameters were significantly associated with other parameters within the same cancer. For adenocarcinoma of the cervix, the age of the patients was significantly associated with tumor size and with depth of infiltration. The histologic grade was strongly associated with presence of lymph node metastasis. FIGO staging was strongly associated with presence of corpus infiltration, vascular invasion, and lymph node metastasis. For adenocarcinoma of endometrium, the age of the patients was associated with menopausal status, FIGO staging with histological grading of the cancer, and presence/absence of myometrial invasion. 

When these clinicopathological parameters were tested against the expression of the three cell cycle markers, none of the clinicopathological parameters of adenocarcinoma of the cervix including the histologic subtypes had significant association with p16INK4a expression alone. However, when p16INK4a expression and p27Kip1 expressions were analyzed in combination, there were strong associations with presence/absence of lymph node metastasis. p21WAF1 expression alone is significantly associated with infiltration of the corpus and lymph node metastasis. Lymph node involvement was also significantly associated with p27Kip1 expression. For adenocarcinoma of endometrium, only P16INK4a expression had strong association with the histologic grade. p21WAF1 and p27Kip1 expressions were not significantly associated with any clinicopathological parameters. Our study shows that we could use these cell cycle markers as predictors for more aggressive subsets of adenocarcinomas of the cervix and endometrium. 

There are a number of studies which have utilized the use of cell cycle regulatory genes such as P16INK4a, p21WAF1, and p27Kip1 in predicting the behavior of certain cancers such as malignant astrocytomas [23], oral squamous cell carcinomas [24], vulval carcinomas [25], and primary large-cell neuroendocrine carcinoma of the parotid gland [26]. In the latter, markedly reduced expressions of p21Waf1 and p27Kip1 were noted in the salivary gland cancer cells indicating highly aggressive biologic behavior [26]. Methylation of p16INK4a was seen to be correlated with gender and tumor size (*P* = 0.005 and *P* = 0.035, resp.) in colorectal carcinoma (CRC) and could be used as a marker of poor prognosis in CRC [27]. In 224 vulvar squamous cell carcinomas stained with p16, p21, and p27, Knopp et al., noted a high expression of p16 as indicator of a better prognosis in the multivariate analysis (RR = 0.5, 95% CI = 0.2–1.0) and less risk of developing lymph node metastasis (OR = 0.3, 95% CI = 0.2–0.7) [25]. They also noted that a high level of p21 was significantly associated with shorter survival in patients staged FIGO I and II (RR = 3.4, 95% CI = 1.3–9.3). We could not find any published literature on utilizing the use of these markers on adenocarcinoma of the cervix and endometrium

In conclusion, for adenocarcinoma of the cervix, p21WAF1 expression is significantly associated with infiltration of the corpus and lymph node metastasis. p27Kip1 expression is significantly associated with lymph node invasion. The presence of lymph node metastasis is strongly associated when p16INK4a and p27Kip1 expressions are analyzed in combination. For adenocarcinoma of the endometrium, p16INK4a expression is associated with histologic grade but not histologic type. Our study shows that we could use these cell cycle markers as predictors for more aggressive subsets of adenocarcinoma of the cervix and endometrium. 

## Figures and Tables

**Table 1:** Summary of the clinicopathological parameters in adenocarcinoma of the cervix (ECA) and adenocarcinoma of the endometrium (EMA).

Variables of ECA	Frequency (%) of ECA	Variables of EMA	Frequency (%) of EMA
Age (years)		Age (years)	
<35	3 (7.5)	<50	28 (30.4)
35–54	22 (55.0)	50–60	35 (38.0)
≥55	15 (37.5)	>60	29 (31.5)

FIGO stage		FIGO stage	
I	27 (57.5)	I	50 (54.3)
ll	7 (17.5)	II	13 (14.1)
lll	5 ((12.5)	lll	13 (14.1)
IV	5 (12.5)	lV	16 (17.4)

Histology tumor differentiation		Myometrial invasion	
Well/moderate	25 (62.5)	<1/2	45 (48.9)
Poor and Special type	15 (37.5)	>1/2	47 (51.1)

Infiltration depth		Histologic grade	
Inner 2/3	13 (32.5)	G1	39 (42.4)
Outer 1/3 or through	27 (67.5)	G2	23 (25.0)
		G3	30 (32.6)

Tumor length		Postmenopausal status	
<20 mm	11 (32.5)	Yes	54 (58.7)
>20 mm	29 (67.5)	No	38 (41.3)

Tumor thickness			
<5 mm	4 (10.0)		
>5 mm	36 (90.0)		

Infiltration to corpus			
Yes	17 (42.5)		
No	23 (57.5)		

Vascular invasion			
Yes	20 (50.0)		
No	20 (50.0)		

Lymph node invasion			
Yes	12 (30.0)		
No	28 (70.0)		

Histologic type:		Histologic type:	
Endocervical NOS	25 (62.5)	Endometrioid	75 (81.5)
Adenosquamous	6 (15.0)	Adenosquamous	4 (4.4)
Endometrioid	5 (12.5)	Clear cell	5 (5.4)
Serous	1 (2.5)	Serous	5 (5.4)
“Mixed”	2 (5.0)	“Mixed”	3 (3.3)

**Table 2:** Interrelationship* between clinicopathologic parameters in patients with adenocarcinoma of the cervix (ECA).

	Age group (*P* value)	Ethnic (*P* value)	Diagnosis (*P* value)	Histo grade (*P* value)	FIGO stage (*P* value)	Infiltration depth (*P* value)	Tumour size (diameter) (*P* value)	Tumor thickness (*P* value)	Corpus infiltration (*P* value)	Vascular invasion (*P* value)	LN invasion (*P* value)
Age group	—	0.140	0.377	0.372	0.582	0.392	**0.006**	0.364	0.301	0.165	0.056
Ethnic		—	0.090	0.850	0.206	0.559	0.838	0.224	0.858	0.140	**0.017**
Histologic type			—	**<0.0001**	0.572	0.191	0.411	0.586	0.804	0.744	0.722
Histologic grade				—	0.090	0.542	0.120	0.586	0.680	0.102	**0.012**
FIGO stage					—	0.330	0.152	1.000	**0.042**	**0.003**	**<0.0001**
Infiltration depth						—	**0.010**	0.056	0.085	**0.018**	0.507
Tumor length							—	**0.025**	0.230	**0.013**	0.076
Tumor thickness								—	0.749	0.292	0.818
Corpus infiltration									—	0.337	**0.043**
Vascular invasion										—	**0.038**
LN invasion											—

*Calculated using Chi-square.

**Table 3:** Interrelationship* between clinicopathological parameters in patients with adenocarcinoma of endometrium (EMA).

	Age group (*P* value)	Menopausal status (*P* value)	Ethnic (*P* value)	Diagnosis (*P* value)	Histology grade (*P* value)	Myometrial invasion (*P* value)	FIGO stage (*P* value)
Age group	—	**<0.0001**	0.139	0.178	0.175	0.101	0.343
Meno status	—	—	0.194	0.270	0.641	0.062	0.260
Ethnic	—	—	—	0.825	0.703	0.248	0.026
Histologic type	—	—	—	—	**<0.0001**	0.213	0.081
Histologic grade	—	—	—	—	—	0.060	**<0.0001**
Myometrial invasion	—	—	—	—	—	—	**<0.0001**
FIGO stage	—	—	—	—	—	—	—

*Calculated using Chi-square.

**Table 4:** Associations between p16INK4a expression and clinicopathologic parameter for endocervical adenocarcinoma.

Characteristics	No. of patients	Positive, *n* (%) (*n* = 32)	Negative, *n* (%) (*n* = 8)	*P* value
Age (years)				
<35	3	2 (66.7)	1 (33.3)	0.256
35–54	22	16 (72.7)	6 (27.3)	
≥55	15	14 (93.3)	1 (6.7)	

FIGO stage				
I-II	30	24 (80.0)	6 (20.0)	1.000
III-IV	10	8 (80.0)	2 (20.0)	

Histology tumor differentiation				
Well/moderate	25	18 (72.0)	7 (28.0)	0.102
Poor	15	14 (93.3)	1 (6.7)	

Infiltration depth				
Inner 2/3	13	9 (69.2)	4 (30.8)	0.237
Outer 1/3 or through	27	23 (85.2)	4 (14.8)	

Tumor size ([diameter)				
<20 mm	11	8 (72.7)	3 (27.3)	0.479
>20 mm	29	24 (82.8)	5 (17.2)	

Tumor thickness				
<5 mm	4	3 (75.0)	1 (25.0)	0.792
>5 mm	36	29 (80.6)	7 (19.4)	

Infiltration to corpus				
Yes	17	13 (76.5)	4 (23.5)	0.631
No	23	19 (82.6)	4 (17.4)	

Vascular invasion				
Yes	20	17 (85.0)	3 (15.0)	0.429
No	20	15 (75.0)	5 (25.0)	

Lymph node invasion				
Yes	12	8 ( 66.7)	4 (33.3)	0.168
No	28	24 (85.7)	4 (14.3)	

Histologic type				
Endocervical	25	20 (80.0)	5 (20.0)	1.000
Non endocervical	15	12 (80.0)	3 (20.0)	

**Table 5:** Associations between P21waf1 expression and clinicopathologic parameter for adenocarcinoma of the cervix.

Characteristics	No. of patients	Positive, *n* (%) (*n* = 28)	Negative, *n* (%) (*n* = 12)	*P* value
Age (years)				
<35	3	2 (66.7)	1(33.3)	0.937
35–54	22	15 (68.1)	7 (31.8)	
≥55	15	11 (73.3)	4 (33.7)	

FIGO stage				
I-II	30	23 (76.7)	7 (23.3)	0.111
III-IV	10	5 (50.0)	5 (50.0)	

Histology tumor differentiation				
Well/moderate	25	16 (64.0)	9 (36.0)	0.285
Poor	15	12 (80.0)	3 (20.0)	

Infiltration depth				
Inner 2/3	13	10 (76.9)	3 (23.1)	0.507
Outer 1/3 or through	27	18 (66.7)	9 (33.3)	

Tumor length				
<20 mm	11	7 (63.6)	4 (36.4)	0.589
>20 mm	29	21 (72.4)	8 (27.6)	

Tumor thickness				
<5 mm	4	1 (25.0)	3 (75.0)	0.101
>5 mm	36	27 (75.0)	9 (25.0)	

Infiltration to corpus				
Yes	17	9 (52.9)	8 (47.1)	**0.043**
No	23	19 (82.6)	4 (17.4)	

Vascular invasion				
Yes	20	15 (75.0)	5 (25.0)	0.490
No	20	13 (65.0)	7 (35.0)	

Lymph node invasion				
Yes	12	6 (50.0)	6 (50.0)	**0.071**
No	28	22 (78.6)	6 (21.4)	

Histologic type				
Endocervical	25	17 (68.0)	8 (32.0)	0.722
Non endocervical	15	11 (73.3)	4 (26.7)	

**Table 6:** Associations between p27Kip1 expression and clinicopathologic parameter for endocervical adenocarcinoma.

Characteristics	No. of patients	Positive, *n* (%) (*n* = 17)	Negative, *n* (%) (*n* = 23)	*P* value
Age (years)				
<35	3	2 (66.7)	1 (33.3)	0.292
35–54	22	7 (31.8)	15 (68.2)	
≥55	15	8 (53.3)	7 (46.7)	

FIGO stage				
I-II	30	15 (50.0)	15 (50.0)	0.097
III-IV	10	2 (20.0)	8 (80.0)	

Histology tumor differentiation				
Well/moderate	25	11 (44.0)	14 (56.0)	0.804
Poor	15	6 (40.0)	9 (60.0)	

Infiltration depth				
Inner 2/3	13	5 (38.5)	8 (61.5)	0.720
Outer 1/3 or through	27	12 (44.4)	15 (55.6)	

Tumor length				
<20 mm	11	5 (45.5)	6 (54.5)	0.818
>20 mm	29	12 (41.4)	17 (58.6)	

Tumor thickness				
<5 mm	4	1 (25.0)	3 (75.0)	0.395
>5 mm	36	16 (44.4)	20 (55.6)	

Infiltration to corpus				
Yes	17	6 (35.3)	11 (64.7)	0.428
No	23	11 (47.8)	12 (52.2)	

Vascular invasion				
Yes	20	7 (35.0)	13 (65.0)	0.337
No	20	10 (50.0)	10 (50.0)	

Lymph node invasion				
Yes	12	2 (16.7)	10 (83.3)	**0.030**
No	28	15 (53.6)	13 (46.4)	

Histologic type				
Endocervical	25	9 (36.0)	16 (64.0)	0.283
Non endocervical	15	8 (53.3)	7 (46.7)	

**Table 7:** Associations between P16INK4a expression and clinicopathologic parameter for endometrial adenocarcinoma.

Characteristics	No. patients	Positive, *n* (%) (*n* = 23)	Negative, *n* (%) (*n* = 69)	*P* value
Age (years)				
<50	28	5 (17.9)	23 (82.1)	0.148
50–60	35	7 ( 20.0)	28 (80.0)	
>60	29	11 (38.0)	18 (62.0)	

FIGO stage				
I	50	12 (24.0)	38 (76.0)	0.809
II–IV	42	11 (26.2)	31 (73.8)	

Myometrial invasion				
<1/2	45	9 (20.0)	36 (80.0)	0.278
>1/2	47	14 (29.8)	33 (70.2)	

Histologic grade				
G1-G2	62	11 (17.7)	51 (82.2)	**0.014**
G3	30	12 (40.0)	18 (60.0)	

Postmenopausal status				
Yes	54	16 (29.6)	38 (70.4)	0.222
No	38	7 (18.4)	31 (81.6)	

Histologic type				
Endometrioid	75	16 (21.3)	59 (78.7)	0.888
Non endometrioid	17	7 (41.2)	10 (58.8)	
